# Supplementary material for: Reversed evolution of grazer resistance to cyanobacteria
Source: Nat Commun. 2021 Mar 29;12:1945. doi: 10.1038/s41467-021-22226-9 (PMC8007715; doi:10.1038/s41467-021-22226-9)
Supplement: Supplementary file 1 — Supplementary Information [file 41467_2021_22226_MOESM1_ESM.pdf]

Supplementary Information

REVERSED EVOLUTION OF GRAZER RESISTANCE TO CYANOBACTERIA

Jana Isanta-Navarro, Nelson G. Hairston Jr, Jannik Beninde, Axel Meyer, Dietmar Straile, Markus Möst and Dominik Martin-Creuzburg

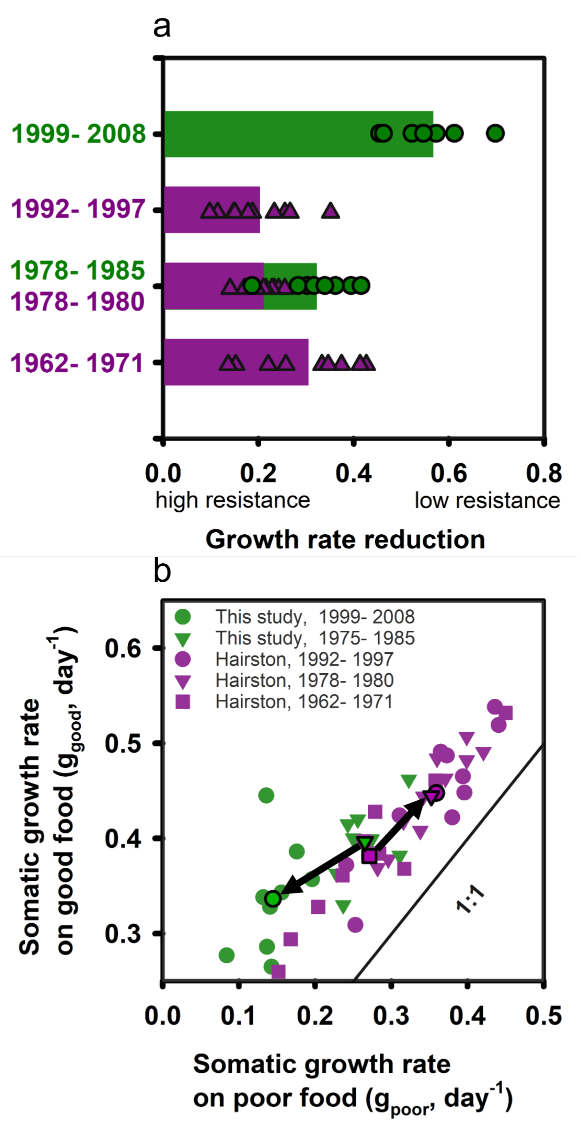

Fig. S1 Performance of *Daphnia* genotypes : Non-standardized data for comparison with Fig. 1a and c (see method section). (a) Resistance of *Daphnia* genotypes to toxic cyanobacteria. Single data points depict average growth rate reduction for individual genotypes, bars the average growth rate reduction of all genotypes within a single time period. For comparison, data from Hairston et al.[1] (purple bars) are included, showing the rapid adaptive evolution of *Daphnia* genotypes to cyanobacteria. (b) Growth rates on good food always exceeded those on poor food. Average juvenile somatic growth rates of genotypes from five time periods are depicted in purple [1] and green (this study) symbols. Evolution of mean juvenile growth rate moved parallel to the 1:1 line over all time intervals but the trajectories of evolution are opposed (black arrows). Source data are provided as a Source Data file.

Rates of Evolution:

Rates of evolution (based on juvenile growth rates) were expressed as haldanes (h), which are defined as the change in mean phenotype in units of standard deviations per generation:

$$h = \frac{\left[\left(\frac{x^2}{s_p}\right)-\left(\frac{x^1}{s_p}\right)\right]}{g} \qquad (1)$$

where x^1 and x^2 are the trait means at the start and the end of the observed time period and g is the number of generations that occurred during that period (assumed here to be one generation, i.e. one sexual reproduction cycle per year), s_p is the pooled standard deviation of the trait at the start and the end of the time period, calculated using the equation:

$$s_p = \sqrt{\frac{(SD_1^2+SD_2^2)}{2}} \qquad (2)$$

where SD_1 and SD_2 are the average standard deviations at the start and the end of the observed time period.

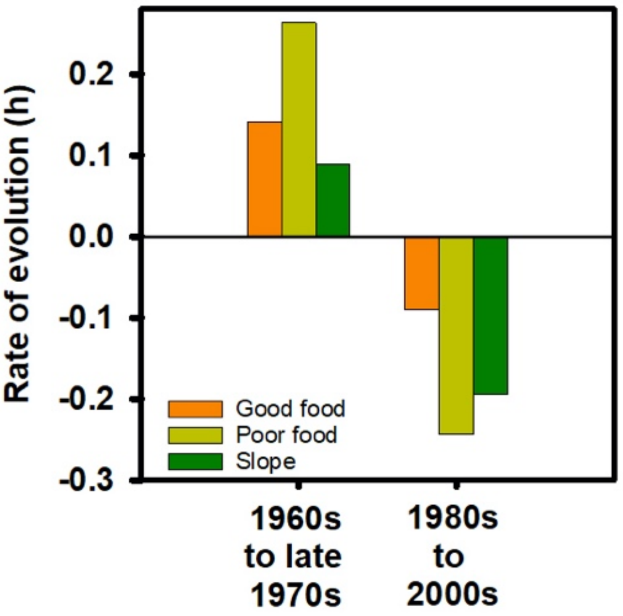

Fig. S2 Rates of evolution: Quantifying rates of trait change provides a comparison of adaptive responses to selection and its reversal. Here we show rates of evolution expressed as haldanes (standard deviations per generation). Rates are shown for (log) juvenile somatic growth rate differences in the two different food environments (orange and ochre) and for the slopes (dark green) of the reaction norms connecting them. Rates of evolution from 1960s to late 1970s are shown for comparison (recalculated from Hairston and De Meester [2]). The decrease in slope of the reaction norm and the increase in juvenile growth rates on poor food from the 1960s to the late 1970s is reflected in positive rates of evolution during eutrophication (adaptive evolution) and the increase in slope and decrease in juvenile growth rate on poor food from 1980s to the 2000s is reflected in negative rates of evolution during oligotrophication (reversed adaptive evolution).The rates of evolution presented here fall well within the range of values reported previously for *Daphnia* and other species [2,3]. Rates of evolution were much faster in the poor than in the good food environment reflecting the high selection pressure imposed by cyanobacteria. Assuming one sexual reproduction cycle per year, for this natural *Daphnia* population in a large temperate lake, it took a maximum of 14-30 generations for the trait to be lost. Relaxation of a selection pressure does not automatically lead to the loss of a trait, and the time of trait decay depends, for instance, on the genetic architecture of the trait, costs involved in maintaining the trait, and the complexity of co-evolved adaptive networks. Source data are provided as a Source Data file.

Ecological and evolutionary components:

The Price Reaction Norm (PRN) approach, combining the strengths of the Price-equation and the reaction norm metrics [4,5], was applied to separate the observed trait changes $\Delta\bar{z}$ into ecological and evolutionary components [6]:

$$\Delta\bar{z} = \sum_{j=1}^{N} z_{22}^{j}(q_2^j - q_1^j) - \sum_{j=1}^{N} q_1^j(z_{21}^j - z_{11}^j) + \sum_{j=1}^{N} q_1^j([z_{22}^j - z_{21}^j] - [z_{12}^j - z_{11}^j]) + \sum_{j=1}^{N} q_1^j(z_{12}^j - z_{11}^j) \qquad (3)$$

where q_1^j (resp. q_2^j) is the relative abundance of genetic lineage *j* at time point t_1 (resp. t_2) and z_{kl}^j is the average trait value of genetic lineage *j* at genetic state *k* (i.e., the population sampled at t_k) and environmental state *l*. The first term captures the change due to lineage sorting, the second term the heritable trait change within lineage, the third term the trait change within lineages due to evolution of plasticity, and the last term the plasticity component. We could not consider trait change due to lineage sorting, because all resurrected clones, which were the product of sexual reproduction, are by definition new lineages, i.e., the relative abundance (q_1^j resp. q_2^j) of genetic lineages at both time points (t_1 resp. t_2) equals 1. Hence, the two processes described here (adaptive evolution and reversed adaptive evolution) were decomposed into only three components: (1) ancestral phenotypic plasticity, (2) evolution of plasticity, and (3) genetic trait change within the population (constitutive evolution). Each term produces an absolute value for the observed trait change that then can be expressed as relative contributions (%) to total trait change. Note that partitioning metrics applied to complex systems are very data-hungry and assessing statistically significant differences is thus challenging [7]. Statistical analyses of the results we report here failed to document significant differences in the relative contribution of the different components between the two transitions. We nevertheless find this partitioning insightful.

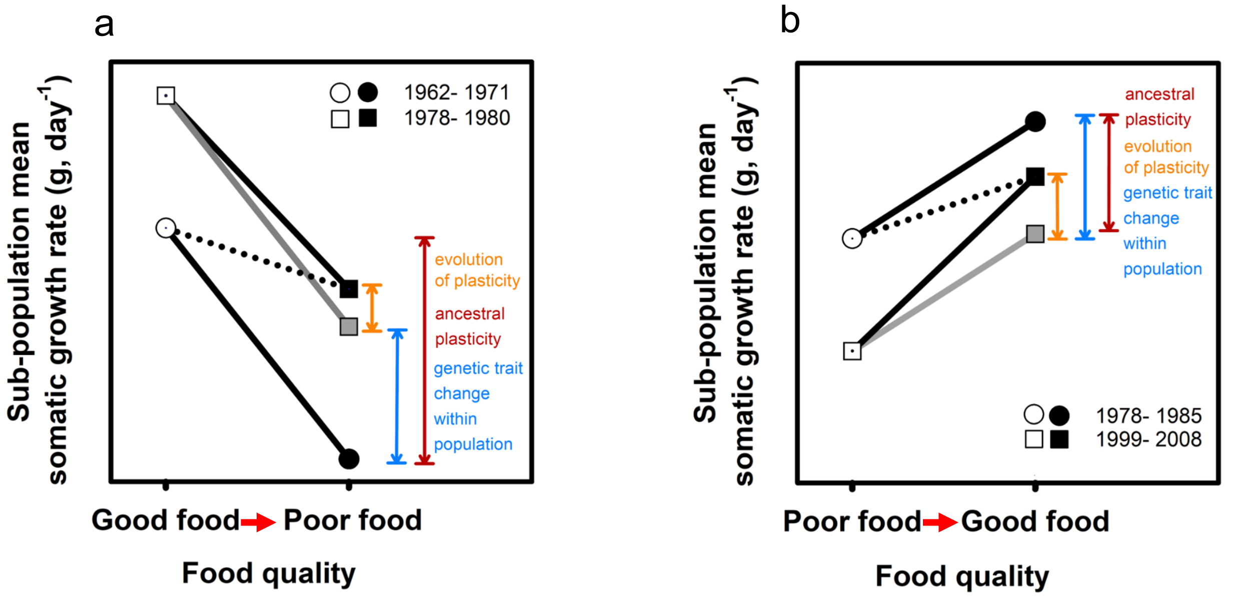

Fig. S3 Price Reaction Norm approach: Univariate reaction norms used to decompose the total trait change during (a) selection imposed by increasing cyanobacteria (eutrophication) and (b) reversed selection imposed by decreasing cyanobacteria abundance (oligotrophication). Red arrows on x-axes show direction of environmental change. Depicted are three components: ancestral plasticity (dark red), evolution of plasticity (orange), and genetic trait change within the population (blue). Open symbols show average juvenile somatic growth rates on food conditions representing the start of the period of consideration (good food for adaptive evolution and poor food for reversed adaptive evolution), filled symbols show juvenile somatic growth rates on food conditions representing the end of the period of consideration (poor food for adaptive evolution, good food for reversed adaptive evolution). Solid black lines represent growth rate reaction norms covering the start and the end of the period of consideration; grey solid lines represent the hypothetical reaction norm of the adapted population without evolution. Grey symbols show hypothetical average growth rates of the adapted population in the new environment without evolution. Dashed lines represent the reaction norms of the realized growth rate of populations in their respective natural environments. The two sequential evolutionary trajectories described here differ in their underlying eco-evolutionary components. The observed change in juvenile somatic growth rate during eutrophication can be explained primarily by ancestral phenotypic plasticity (57.6 %) combined with genetic trait change within the population (33.0 %), and to a lesser extent by evolution of plasticity (9.4 %). In contrast, the trait change during oligotrophication was characterized by a lower contribution of ancestral phenotypic plasticity (41.1 %) and a higher contribution of evolution of plasticity (19.7 %), while the genetic trait change within the population was similar (39.2 %). Source data are provided as a Source Data file.

References in Supplementary Information

1. Hairston, N. G., Jr. *et al.* Lake ecosystems: rapid evolution revealed by dormant eggs. *Nature* **401**, 446 (1999).
2. Hairston, N. G., Jr. & De Meester, L. *Daphnia* paleogenetics and environmental change: deconstructing the evolution of plasticity. *Int. Rev. Hydrobiol.* **93**, 578–592 (2008).
3. Hendry, A. P. & Kinnison, M. T. Perspective: The pace of modern life: measuring rates of contemporary microevolution. *Evolution* **53**, 1637–1653 (1999).
4. Price, G. R. Selection and covariance. *Nature* **227**, 520–521 (1970).
5. Ellner, S. P., Geber, M. A. & Hairston, N. G., Jr. Does rapid evolution matter? Measuring the rate of contemporary evolution and its impacts on ecological dynamics. *Ecol. Lett.* **14**, 603–614 (2011).
6. Govaert, L., Pantel, J. H. & De Meester, L. Eco-evolutionary partitioning metrics: assessing the importance of ecological and evolutionary contributions to population and community change. *Ecol. Lett.* **19**, 839–853 (2016).
7. Govaert, L. Eco-evolutionary partitioning metrics: a practical guide for biologists. *Belg. J. Zool.* **148**, 167–202 (2018).
