# Supplementary material for: Reversed evolution of grazer resistance to cyanobacteria
Source: Nat Commun. 2021 Mar 29;12:1945. doi: 10.1038/s41467-021-22226-9 (PMC8007715; doi:10.1038/s41467-021-22226-9)
Supplement: Supplementary file 3 — Reporting Summary [file 41467_2021_22226_MOESM3_ESM.pdf]

nature research

Corresponding author(s): Jana Isanta-Navarro

Last updated by author(s): 2021/02/17

Reporting Summary

Nature Research wishes to improve the reproducibility of the work that we publish. This form provides structure for consistency and transparency in reporting. For further information on Nature Research policies, see our Editorial Policies and the Editorial Policy Checklist.

Statistics

For all statistical analyses, confirm that the following items are present in the figure legend, table legend, main text, or Methods section.

n/a	Confirmed	
☐	☒	The exact sample size (*n*) for each experimental group/condition, given as a discrete number and unit of measurement
☐	☒	A statement on whether measurements were taken from distinct samples or whether the same sample was measured repeatedly
☐	☒	The statistical test(s) used AND whether they are one- or two-sided<br>*Only common tests should be described solely by name; describe more complex techniques in the Methods section.*
☐	☒	A description of all covariates tested
☐	☒	A description of any assumptions or corrections, such as tests of normality and adjustment for multiple comparisons
☐	☒	A full description of the statistical parameters including central tendency (e.g. means) or other basic estimates (e.g. regression coefficient) AND variation (e.g. standard deviation) or associated estimates of uncertainty (e.g. confidence intervals)
☐	☒	For null hypothesis testing, the test statistic (e.g. *F*, *t*, *r*) with confidence intervals, effect sizes, degrees of freedom and *P* value noted<br>*Give P values as exact values whenever suitable.*
☒	☐	For Bayesian analysis, information on the choice of priors and Markov chain Monte Carlo settings
☒	☐	For hierarchical and complex designs, identification of the appropriate level for tests and full reporting of outcomes
☒	☐	Estimates of effect sizes (e.g. Cohen's *d*, Pearson's *r*), indicating how they were calculated

Our web collection on statistics for biologists contains articles on many of the points above.

Software and code

Policy information about availability of computer code

Data collection	no Software was used
Data analysis	SigmaPlot, Systat Software, Version 14.0. , STRand Version 2.4.110

For manuscripts utilizing custom algorithms or software that are central to the research but not yet described in published literature, software must be made available to editors and reviewers. We strongly encourage code deposition in a community repository (e.g. GitHub). See the Nature Research guidelines for submitting code & software for further information.

Data

Policy information about availability of data

All manuscripts must include a data availability statement. This statement should provide the following information, where applicable:

- Accession codes, unique identifiers, or web links for publicly available datasets
- A list of figures that have associated raw data
- A description of any restrictions on data availability

The data that support the findings of this study are available from the corresponding author upon reasonable request.

Field-specific reporting

Please select the one below that is the best fit for your research. If you are not sure, read the appropriate sections before making your selection.

☐ Life sciences ☐ Behavioural & social sciences ☒ Ecological, evolutionary & environmental sciences

For a reference copy of the document with all sections, see nature.com/documents/nr-reporting-summary-flat.pdf

Ecological, evolutionary & environmental sciences study design

All studies must disclose on these points even when the disclosure is negative.

Study description	We explored experimentally how the capacity of Daphnia to cope with cyanobacterial food changed with oligotrophication of Lake Constance. Life-history experiments were conducted with Daphnia galeata genotypes resurrected from diapausing eggs that were isolated from dated sediment layers representing peak-eutrophic (hereafter 1980s) and re-oligotrophic conditions (hereafter 2000s). The data obtained are compared with previous results showing the adaptive evolution of resistance using genotypes from pre-eutrophic (hereafter 1960s) and peak-eutrophic conditions. This allowed us to document trait changes over almost half a century and to disentangle the different ecological and evolutionary components mediating the evolution of resistance to cyanobacteria.
Research sample	Genotypes from two different time periods were resurrected. The genotypes from each time period are supposed to approximate the population during two different trophic states of Lake Constance. The resurrection approach is the only possibility to obtain genotypes from past decades. Female Daphnia were used for the experiment, that were <12h old when the experiment started.
Sampling strategy	experimental set-up was oriented on previously published experiments (Hairston et al.,1999, 2001).
Data collection	A fully randomized life-history experiment was performed to collect data. The experiment was performed by Jana Isanta-Navarro, individuals were observed daily to record life-history parameters.
Timing and spatial scale	The experiment started 02/08/2019 and was terminated after all individuals either died or reproduced 3 times. Individuals were checked and transferred daily to keep the food quantity and quality constant. Life-history parameters (juvenile somatic growth rate, mortality, age at reproduction, number of offspring) were recorded as they represent a valid approximation for fitness.
Data exclusions	none excluded
Reproducibility	The experimental approach and the applied data analyses are clearly described in the Method section and the isolated Daphnia clones that were used here are still available, ensuring reproducibility of experimental findings. The two attempts to repeat the experiment were successful and revealed similar results.
Randomization	Daphnia were randomly assigned to the experimental treatments; grouped according to the year they were resurrected from
Blinding	The organisms used in this experiment are incapable of recognizing the experimental set-up and purpose of the study. The experimental set-up did not allow for blinding of treatments because the food suspensions had to be renewed daily.

Did the study involve field work? ☐ Yes ☒ No

Reporting for specific materials, systems and methods

We require information from authors about some types of materials, experimental systems and methods used in many studies. Here, indicate whether each material, system or method listed is relevant to your study. If you are not sure if a list item applies to your research, read the appropriate section before selecting a response.

Materials & experimental systems

n/a	Involved in the study	
☒	☐	Antibodies
☒	☐	Eukaryotic cell lines
☒	☐	Palaeontology and archaeology
☐	☒	Animals and other organisms
☒	☐	Human research participants
☒	☐	Clinical data
☒	☐	Dual use research of concern

Methods

n/a	Involved in the study	
☒	☐	ChIP-seq
☒	☐	Flow cytometry
☒	☐	MRI-based neuroimaging

Animals and other organisms

Policy information about studies involving animals; ARRIVE guidelines recommended for reporting animal research

Laboratory animals	resurrected Daphnia galeata genotypes
Wild animals	none
Field-collected samples	ephippia: Sediment cores were taken in spring 2017 in the Bay of Friedrichshafen in Upper Lake Constance (47°36´26.51″N 9°27´45.82″E) using a multicorer 47. A total of 21 sediment cores were taken; three sediment cores at a time. The cores were split vertically into halves using a metal sheet and stored at 4 °C in the dark. Sediment cores were partitioned into centimeter sections, reflecting the time period from 1945 to 2010. The age of the different sediment layers was determined according to Wessels et al. 48. The sediment samples were sieved through a 250 µm mesh and ephippia (protective chitinous shells normally containing two diapausing eggs) were isolated using a stereomicroscope. Ephippia were directly transferred into 24-well-plates containing filtered water (<0.2 µm) from Lake Constance and exposed to artificial daylight (16:8 L:D) and room temperature (20 °C) to stimulate egg hatching.
Ethics oversight	not applicable (invertebrates)

Note that full information on the approval of the study protocol must also be provided in the manuscript.
